# Psychosocial response to the COVID-19 pandemic in Panama

**DOI:** 10.3389/fpubh.2022.919818

**Published:** 2022-08-10

**Authors:** Diana C. Oviedo, María Sofía Pinzón, Sofía Rodríguez-Araña, Adam E. Tratner, Elianne Pauli-Quirós, Carlos Chavarría, Camilo Posada Rodríguez, Gabrielle B. Britton

**Affiliations:** ^1^Centro de Neurociencias y Unidad de Investigación Clínica, Instituto de Investigaciones Científicas y Servicios de Alta Tecnología (INDICASAT-AIP), Panama City, Panama; ^2^Escuela de Psicología, Universidad Santa María la Antigua (USMA), Panama City, Panama; ^3^Sistema Nacional de Investigación (SNI) SENACYT, Panama City, Panama; ^4^SENACYT, Florida State University, Panama City, Panama

**Keywords:** COVID−19, psychological distress, mental health, restrictions, depression, anxiety, stress

## Abstract

**Background:**

The impact of the COVID-19 pandemic and the associated restrictions on mental health is being studied.

**Objective:**

To analyze the psychosocial response to the COVID-19 pandemic in adults residing in Panama.

**Methods:**

A community sample of 480 adult residents of Panama completed a survey that included sociodemographic questions, COVID-19 related questions (e.g., health concerns regarding the virus, knowledge and behaviors in biosafety) and scales of stress, anxiety, depression, prosocial behavior, resilience, perceived social support, and insomnia.

**Results:**

Most of the participants (>60%) reported being negatively affected by the pandemic. Women experienced greater depression, anxiety, and stress symptoms than men, and age was negatively associated with depression, anxiety, and stress symptoms. Self-perceived health status and self-perceived social support were negatively associated with depression, anxiety, and stress symptoms. Self-perceived social isolation was positively associated with depression, anxiety, and stress symptoms. Psychiatric illness and insomnia were positively associated with depression, anxiety, and stress symptoms, whereas psychological resilience was negatively associated with depression, anxiety, and stress symptoms.

**Discussion:**

These results corroborate other studies regarding COVID-19 and mental health. This study highlights the need for specific prevention and intervention mechanisms related to the COVID-19 pandemic in different population groups. This is the first report of the psychological impact of COVID-19 in the general Panamanian population and one of the only studies in the Latin American region and, therefore, contributes to research in the Latino population and lower-middle income countries.

## Introduction

By early 2020, the SARS-CoV-2 coronavirus disease 2019 (COVID-19) had spread rapidly throughout the world and was officially declared a pandemic in March 2020. To prevent the propagation of the virus, many countries adopted different mitigation strategies such as quarantines, rigorous lockdowns, mobility restrictions, closure of schools and the isolation of vulnerable populations (1). Research on previous epidemics, such as Middle East Respiratory Syndrome (MERS) and Severe Acute Respiratory Syndrome (SARS), documented that these measures are associated with an increase in mental health-related distress (2, 3). Studies have reported high-stress levels, sleep disturbances such as insomnia and nightmares, an increase in depressive and anxiety symptoms, and poor concentration, among others (4).

Moreover, research on the effects of COVID-19 lockdowns has documented high anxiety, depression, and stress levels, as well as post-traumatic stress disorder symptoms, irritability, isolation, fear, uncertainty, anger, loneliness, and guilt in people who experienced ongoing restrictions (5–7). These psychological manifestations are associated with personal, social, behavioral and cognitive variables that, taken together, encompass psychosocial determinants of how people respond to menacing situations (8). These social determinants of the pandemic have impacted individual emotional distress (9). Research has shown that the social determinants related to worse psychological responses to the pandemic include being female, age, previous mental health diagnosis, lower income, racial and ethnic disparities, poor subjective and objective health status, and being a healthcare worker (10–13). Furthermore, the rise in psychological and psychiatric symptoms is also a result of COVID-19 related deaths and illnesses as well as social unrest and economic crises (14, 15).

Most research investigating mental health during the pandemic has focused on high-income countries, and there is limited empirical research on COVID-19's psychosocial effects on low and middle-income countries (LMIC), specifically in Latin America (16). For instance, in Panama, the only reported study regarding mental health in healthcare workers, has evidenced a high prevalence of mental health disorders in this population group (17). Panama had one of the strictest lockdown measures in the world (18), which lasted until late 2021 (19). The country implemented various restrictive mechanisms to mitigate and control the spread of the pandemic: mobility and travel restrictions according to ID number and sex, suspension of in-person educational activities and social activities, border closures, sanitary and epidemiological control, staff rotation and teleworking modality (20).

Furthermore, in Panama as well as in other LMIC, the COVID-19 pandemic exposed economic, social, health and educational inequalities that affected the most disadvantaged and vulnerable individuals (21). While many affluent countries have experienced severe health crises, low and middle-income countries have undergone more pronounced economic crises that are projected to continue (22). In developing countries, it is estimated that 255 million full-time jobs have been lost (23). Additionally, the informal sector has been severely impacted by the pandemic. Around 67% of people with informal jobs live in developing countries, and most have been affected by lockdown measures, affecting economic stability (22). Moreover, in developing countries there is a high prevalence of comorbidities, coupled with limited access to health services, particularly mental health resources (24, 25). All these factors increase the toll on the mental health of residents in developing countries. Therefore, this study aims to explore the psychosocial determinants associated with the pandemic in the Panamanian population. The objective of this study was to analyze the psychosocial response to the COVID-19 pandemic in adults residing in Panama.

## Methods

### Participants and procedure

This was a descriptive, quantitative, cross-sectional study. Participants were 480 adult residents of Panama (80.8% women), aged 18 years or older (*M* = 32.7; SD = 14.6, Range = 18–66). Participants were recruited using convenience sampling. Sample size was calculated using Raosoft Sample Size Calculator. Considering 2,958,577 as the population of people 18 years and older in Panama (26), prevalence of psychosocial effects of the pandemic at 30% (average reported psychosocial effects of pandemic in previous studies), at 95% confidence levels and 5% error margin, the estimated minimum sample size was 323. The research team used advertisements on social media platforms (e.g., Instagram, Facebook, Twitter), which included a description of the study and the principal investigator's contact information. Those who voluntarily contacted the principal investigator were provided access to an online survey *via* a Google Forms link if they met the inclusion criteria of being an adult over 18 years old resident of Panama, having access to a technological device such as a laptop, cellphone or tablet, and not having a physical condition that would make it difficult or preclude accessing the link or answering questions (e.g., visual impairment, cognitive impairment, illiteracy). Recruitment and data collection took place from March 26, 2021 to May 11, 2021. This study was approved by the National Research Bioethics Committee of Panama (CNBI). Participants provided informed consent in compliance with the Declaration of Helsinki principles (1964). The online survey consisted of sociodemographic questions regarding sex, age, marital status, the number of cohabitants living in the same household, employment status, and monthly income. Participants also indicated how many chronic illnesses (e.g., diabetes, hypertension) and psychiatric disorders (e.g., depression, anxiety) they had been diagnosed with, as well as their self-perceived health status (0 = Very bad, 2 = Average, 4 = Very good). In addition, questions assessed biosafety knowledge and behaviors, as well as attitudes and health concerns pertaining to COVID-19. Lastly, several scales that measure psychological symptoms and manifestations linked to COVID-19 pandemic outcomes were included.

### Measures

The Depression, Anxiety and Stress Scale-21 (DASS-21) (27) was used to report symptoms of depression, anxiety, and stress. The Athens Scale of Insomnia (ASI) (28, 29) was included to indicate if participants experienced sleep difficulties at least 3 times in the past month and the severity of their symptoms. Participants also completed a self-report measure of prosocial behavior (Prosociality Scale) (30). Additionally, participants reported perceived psychological resilience during the past month using the Connor-Davidson Resilience Scale (CD-RISC) (31). Finally, the Multidimensional Scale of Perceived Social Support (MSPSS) (32) was included to assess the perceived quality of social support from family, friends, and relationship partners.

## Results

### Statistical analyses

Statistical analyses were conducted using IBM SPSS Statistics version 27.0. Descriptive statistics were used to summarize the demographic characteristics of the sample. Means and standard deviations were calculated for quantitative variables, and categorical variables were presented as frequencies and percentages. Univariate analyses were used to compare groups and examine relationships between variables of interest. Specifically, we used analysis of variance to investigate sex and age cohort differences, and hierarchical linear regression to examine the unique contribution of demographic, economic, health, social psychological, and psychiatric factors on psychological distress symptoms. Results for which *p* < 0.05 were accepted as significant.

### Results

Table 1 summarizes the sample's sociodemographic characteristics. The majority of participants were Panamanian nationals (88.5%), single (79.4%), educated (76.1% completed a bachelor's degree or higher), female (80.8%), and cohabitated with one or more people (94.4%). More than one-third (37.3%) of participants were unemployed at the time of the survey, and less than half of the sample (46.3%) earned a monthly income higher than $2,000.

**Table 1:** Sociodemographic characteristics.

	**Total (*N* = 480)**	**Female (*N* = 388)**	**Male (*N* = 92)**
	***n* (%)/M (SD)**	***n* (%)/M (SD)**	***n* (%)/M (SD)**
**Sex**			
Female	388 (80.8%)	-	-
Male	92 (19.2%)	-	-
Age	32.7 (14.6)	32.4 (14.3)	33.8 (15.6)
**Nationality**			
Panamanian	425 (88.5%)	344 (88.7%)	81 (88.0%)
Other	55 (11.5%)	44 (11.3%)	11 (12.0%)
**Marital status**			
Married/Partnered	99 (20.6%)	77 (19.8%)	22 (23.9%)
Single/Divorced/ Widowed	381 (79.4%)	311 (80.2%)	70 (76.1%)
**Education level**			
High school diploma	66 (13.8%)	48 (12.4%)	18 (19.6%)
Bachelor's degree	235 (49.0%)	189 (48.7%)	46 (50.0%)
Graduate degree	130 (27.1%)	110 (28.4%)	20 (21.7%)
**Employment status**			
Unemployed	179 (37.3%)	144 (37.1%)	35 (38.0%)
Independent work	76 (15.8%)	63 (16.2%)	13 (14.1%)
Permanent contract	151 (31.5%)	121 (31.2%)	30 (32.6%)
Other	74 (15.4%)	60 (15.5%)	14 (15.2%)
**Monthly household income**			
$800–$1,500	94 (19.6%)	79 (20.4%)	15 (16.3%)
$1,500–$2,000	83 (17.3%)	65 (16.8%)	18 (19.6%)
> $2,000	222 (46.3%)	177 (45.6%)	45 (48.9%)
Other	81 (16.8%)	67 (17.3%)	14 (15.2%)
**Cohabitation**			
Live alone	27 (5.6%)	21 (5.4%)	6 (6.5%)
2 Cohabitants	111 (23.1%)	89 (22.9%)	22 (23.9%)
3 Cohabitants	116 (24.2%)	93 (24.0%)	23 (25.0%)
4 Cohabitants	126 (26.3%)	103 (26.5%)	23 (25.0%)
5+ Cohabitants	100 (20.8%)	82 (21.1%)	18 (19.6%)

Table 2 shows the perception of risk and health factors. Most participants (79.2%) reported their overall health as “Good” or “Very good,” whereas 26.9% reported having one or more chronic illnesses (e.g., diabetes, hypertension, obesity). Additionally, 21% of participants reported having a psychiatric diagnosis (e.g., depression, anxiety, agoraphobia), and nearly one-third of the sample (32.1%) reported taking at least one prescribed medication. In addition, most participants did not report an increase in cigarette (14.6%) or alcohol (22.1%) consumption. However, most participants (78.3%) reported changes in their amount of physical activity.

**Table 2:** Subjective health and risk factors.

	**Total (*N* = 480)**	**Female (*N* = 388)**	**Male (*N* = 92)**
	***n* (%)/M (SD)**	***n* (%)/M (SD)**	***n* (%)/M (SD)**
**Subjective health**			
Very good	120 (25.0%)	92 (23.7%)	28 (30.4%)
Good	260 (54.2%)	211 (54.4%)	49 (53.3%)
Regular	94 (19.6%)	81 (20.9%)	13 (14.1%)
Poor	5 (1.0%)	4 (1.0%)	1 (1.1%)
Very poor	1 (0.2%)	-	1 (1.1%)
**Participants with Chronic illnesses**			
Yes	129 (26.9%)	99 (25.5%)	30 (32.6%)
Diabetes	14 (2.91%)	44 (11.3%)	11 (12.0%)
Hypertension	45 (9.38%)	77 (19.8%)	22 (23.9%)
Obesity	30 (6.25%)	24 (6.2%)	6 (6.5%)
Arthritis	4 (0.83%)	3 (0.8%)	1 (1.1%)
Cancer	4 (0.83%)	1 (0.3%)	3 (3.3%)
Renal Illness	1 (0.2%)	1 (0.3%)	0 (0%)
Pulmonary Illness	12 (2.5%)	9 (2.3%)	3 (3.3%)
Cardiac Illness	6 (1.3%)	2 (0.5%)	4 (4.3%)
Vascular Illness	2 (0.4%)	2 (0.5%)	0 (0%)
Other Chronic Illness	54 (11.3%)	49 (12.6%)	5 (5.4%)
**Participants with Psychiatric Illnesses**			
Yes	101 (21.0%)	88 (22.7%)	13 (14.1%)
Depression	63 (13.1%)	56 (14.4%)	7 (7.6%)
Anxiety	74 (15.4%)	64 (16.5%)	10 (10.9%)
Schizophrenia	0 (0%)	0 (0%)	0 (0%)
Agoraphobia	1 (0.2%)	1 (0.3%)	0 (0%)
Social Phobia	3 (0.6%)	3 (0.8%)	0 (0%)
Other Psychiatric Illness	22 (4.6%)	18 (4.6%)	4 (4.3%)
**Participant takes at least one medication**			
Yes	154 (32.1%)	121 (31.2%)	33 (35.9%)
**Participant has forgotten or increased his/her dose**			
Frequently	21 (13.9%)	19 (15.7%)	2 (6.6%)
Occasionally	45 (29.8%)	37 (30.6%)	8 (26.7%)
Rarely	85 (56.3%)	65 (53.7%)	20 (66.7%)
**Cigarette consumption**			
Frequently	12 (2.5%)	7 (1.8%)	5 (5.4%)
Occasionally	19 (4.0%)	14 (3.6%)	5 (5.4%)
Never	449 (93.5%)	367 (94.6%)	82 (89.1%)
**Increase in cigarette consumption**			
Yes	14 (14.6%)	9 (12.7%)	5 (20.0%)
**Alcoholic beverage consumption**			
Frequently	49 (10.2%)	35 (9.0%)	14 (15.2%)
Occasionally	210 (43.8%)	168 (43.3%)	42 (45.7%)
Never	221 (46.0%)	185 (47.7%)	36 (39.1%)
**Increase in alcoholic beverage consumption**			
Yes	85 (22.1%)	67 (21.6%)	18 (24.0%)
**Physical activity before the pandemic**			
Frequently	160 (33.3%)	115 (29.6%)	45 (48.9%)
Occasionally	120 (25.0%)	99 (25.5%)	21 (22.8%)
Rarely	200 (41.7%)	174 (44.8%)	26 (28.3%)
**Change in physical activity during the pandemic**			
Yes	376 (78.3%)	306 (78.9%)	70 (76.1%)
**Change in level of physical activity**			
No longer engaged in physical activity	103 (27.9%)	78 (25.9%)	25 (36.7%)
Rarely engaged in physical activity	57 (15.4%)	45 (14.9%)	12 (17.6%)
Engaged in physical activity at least once a week	76 (20.6%)	67 (22.2%)	9 (13.2%)

Many participants reported disturbances to their psychosocial well-being during the pandemic. For instance, 35% of participants reported mild to moderate levels of depression, 25% reported mild to moderate anxiety symptoms, and 51% reported mild to moderate levels of stress. Table 3 summarizes the aspects of participants' lives that were most affected by the pandemic, as well as perceived risk of contagion, social isolation, and the ability to overcome the pandemic. For example, 44.1% of participants reported that they had felt socially isolated from others during confinement. Most participants reported that the areas that were most negatively impacted were recreational activities and hobbies (74.6%), social relationships (67.7%), mental health (62.9%), and the economy (50.4%). Regarding risk of contagion, 12% of participants believed that they are at risk of COVID-19 infection due to having a chronic disease, 9% due to being an older adult, and 7% due to high exposure to the virus at work. Nine percent of participants reported that they are at risk due to being pregnant, immunosuppressed, a smoker, and not following biosecurity measures.

**Table 3:** Contagion risk, affected areas and psychological attention.

	**Total (*N* = 480)**	**Female (*N* = 388)**	**Male (*N* = 92)**
	***n* (%)/M (SD)**	***n* (%)/M (SD)**	***n*(%) / M (SD)**
**Risk of COVID-19 contagion**			
Agree	193 (40.2%)	158 (40.7%)	35 (38.0%)
Unsure	71 (14.8%)	60 (15.5%)	11 (12.0%)
Disagree	216 (45.0%)	170 (43.8%)	46 (50.0%)
**Testing positive for COVID-19**			
I had symptoms and was tested	63 (13.1%)	46 (11.9%)	17 (18.5%)
No, but the people I live with had symptoms or tested positive	62 (12.9%)	49 (12.6%)	13 (14.1%)
No and none of the people I live with presented symptoms	355 (74.0%)	293 (75.5%)	62 (67.4%)
**Close relatives testing positive for COVID-19**			
Relatives and friends	199 (41.5%)	161 (41.5%)	38 (41.3%)
**Close relatives and friends that passed away from COVID-19**			
Family	66 (13.8%)	51 (13.1%)	15 (16.3%)
Friends	73 (15.2%)	60 (15.5%)	13 (14.1%)
**Currently quarantined**			
No	427 (89.0%)	341 (87.9%)	86 (93.5%)
Other	53 (11.0%)	47 (12.1%)	6 (6.5%)
**Lack of companionship**			
Frequently	172 (35.8%)	136 (35.1%)	36 (39.1%)
Occasionally	133 (27.7%)	111 (28.6%)	22 (23.9%)
Rarely	175 (36.5%)	141 (36.3%)	34 (37.0%)
**Emotional isolation**			
Frequently	212 (44.1%)	174 (44.9%)	38 (41.3%)
Occasionally	141 (29.4%)	118 (30.4%)	23 (25.0%)
Rarely	127 (26.5%)	96 (24.7%)	31 (33.7%)
**Able to cope with the pandemic**			
Agree	401 (83.5%)	317 (81.7%)	84 (91.3%)
Unsure	37 (7.7%)	33 (8.5%)	4 (4.3%)
Disagree	42 (8.8%)	38 (9.8%)	4 (4.3%)
**Affected by the COVID-19 pandemic**			
Affected	313 (65.2%)	255 (65.7%)	58 (63.0%)
Slightly affected	167 (34.8%)	133 (34.3%)	34 (37.0%)
**Affected areas**			
Mental health	302 (62.9%)	254 (65.5%)	48 (52.2%)
Economy	242 (50.4%)	195 (50.3%)	47 (51.1%)
Social relations	325 (67.7%)	261 (67.3%)	64 (69.6%)
Recreational activities and hobbies	358 (74.6%)	292 (75.3%)	66 (71.7%)
**Receiving psychological attention currently**			
Yes	96 (20.0%)	82 (21.1%)	14 (15.2%)
**Received psychological attention in the past**			
Yes	249 (51.9%)	214 (55.2%)	35 (38.0%)
**Satisfied with the support of family and friends**			
Agree	390 (81.2%)	313 (80.7%)	77 (83.7%)
Disagree	68 (14.2%)	59 (15.2%)	9 (9.8%)
Unsure	22 (4.8%)	16 (4.1%)	6 (6.5%)

Additionally, approximately one in 10 of those surveyed (11%) were placed under mandatory quarantine (imposed by the government) because they had either tested positive for COVID-19 or were in close contact with someone who had tested positive for the virus. Half of the sample indicated that they frequently received information about the virus, and most of the participants (93%) reported that they knew, complied with, and agreed with the biosafety measures recommended by the Ministry of Health (MINSA). Most participants (93.8%) stated that they complied with biosafety measures because they wanted to take care of their health and that of others, while the rest complied with these measures because they were forced to do so, they were afraid of receiving a fine, or they were afraid of being detained by authorities.

### Analysis of variance

Analysis of variance was used to examine sex differences. There was a significant difference between men and women in depression scores, such that women (*M* = 13.8) had a higher mean score of depression than men (*M* = 10.6), *F*_(1, 479)_ = 4.76, *p* = 0.03. Women (*M* = 10.0) also had higher anxiety scores than men (*M* = 6.8), *F*_(1, 479)_ = 9.48, *p* = 0.002, and higher (*M* = 17.0) stress scores than men (*M* = 13.0), *F*_(1, 479)_ = 12.44, *p* < 0.001. However, there were no sex differences in resilience scores, *F*_(1, 479)_ = 3.73, *p* = 0.054, insomnia scores, *F*_(1, 479)_ = 1.92, *p* = 0.167, perceived social support, *F*_(1, 479)_ = 0.64, *p* = 0.423, or prosociality, *F*_(1, 479)_ = 2.50, *p* = 0.114. In sum, women reported higher depression, anxiety, and stress scores compared to men, but there were no significant differences in resilience, insomnia, prosociality, or perceived social support.

Similarly, analysis of variance was used to examine differences between age groups. Participants were divided into two groups: young adults (18–29 years of age) and adults (aged 30 and older). There was a statistically significant difference between those younger than 29 and those older than 30 years of age in depression scores, such that those younger adults (*M* = 14.5) had significantly higher scores than older adults (*M* = 9.0), [*F*_(1, 478)_ = 44.00, *p* < 0.001. Younger adults (*M* = 11.0) also reported higher anxiety scores than older adults (*M* = 7.1), *F*_(1, 478)_ = 21.49, *p* < 0.001, and higher stress scores (*M* = 18.4) than older adults (*M* = 13.1), *F*_(1, 478)_ = 36.14, *p* < 0.001. Conversely, older adults (*M* = 76.12) reported significantly higher resilience scores than younger adults (*M* = 68.6), *F*_(1, 478)_ = 34.60, *p* < 0.001. Further, older adults (*M* = 47.4) reported higher prosociality scores than younger adults (*M* = 45.7), *F*_(1, 478)_ = 4.01, *p* = 0.046. There was not a significant difference between groups in insomnia scores, *F*_(1, 478)_ = 1.23, *p* = 0.268, or perceived social support scores, *F*_(1, 478)_ = 1.47, *p* = 0.227. In sum, younger adults reported worse depression, anxiety, and stress scores than older adults, while older adults reported higher resilience and prosociality scores than younger adults.

### Hierarchical multiple linear regression

A hierarchical multiple regression analysis was performed to investigate whether sociodemographic characteristics, economic factors, physical health, social factors, and mental health are uniquely related to depression, anxiety, and stress symptoms (Table 4). The composite sum score of all DASS-21 subscales was used as the criterion variable. Predictor variables were entered stepwise: education level, marital status, sex, and age were added as predictor variables in Step 1, monthly income and employment status were added as predictor variables in Step 2, self-perceived health status and the number of diagnosed chronic illnesses were added as predictor variables in Step 3, the composite sum score of all MSPSS subscales, self-perceived loneliness, number of cohabitants, and self-perceived isolation were added as predictors in Step 4, and the total number of diagnosed psychiatric disorders, the composite sum score of all CD-RISC subscales, and the composite sum score of all AIS subscales were added as predictors in Step 5.

**Table 4:** Hierarchical multiple linear regression.

	***b***	**SE *B***	**β**	***t***	***p***
**Step 1**					
Sex	−4.956	1.508	−0.142	−3.287***	0.001
Age	−0.290	0.043	−0.308	−6.708***	0.000
Marital status	−0.400	0.480	−0.036	−0.833	0.405
Education level	−0.422	0.555	−0.035	−0.762	0.447
**Step 2**					
Sex	−4.673	1.518	−0.134	−3.079**	0.002
Age	−0.294	0.048	−0.313	−6.130***	0.000
Marital status	−0.457	0.482	−0.041	−0.948	0.344
Education level	−0.177	0.576	−0.015	−0.307	0.759
Monthly income	−2.440	1.561	−0.088	−1.563	0.119
Employment status	0.462	0.675	0.041	0.684	0.494
**Step 3**					
Sex	−3.935	1.453	−0.113	−2.708**	0.007
Age	−0.334	0.050	−0.354	−6.723***	0.000
Marital status	−0.587	0.460	−0.053	−1.277	0.202
Education level	0.215	0.551	0.018	0.390	0.697
Monthly income	−1.693	1.490	−0.061	−1.136	0.257
Employment status	0.324	0.643	0.029	0.504	0.615
Perceived health	−5.738	0.849	−0.293	−6.760***	0.000
Chronic illness	0.341	0.964	0.017	0.354	0.724
**Step 4**					
Sex	−3.332	1.137	−0.096	−2.929**	0.004
Age	−0.141	0.041	−0.150	−3.488***	0.001
Marital status	−0.407	0.366	−0.037	−1.112	0.267
Education level	0.320	0.430	0.026	0.745	0.457
Monthly income	−0.069	1.171	−0.003	−0.059	0.953
Employment status	−0.460	0.503	−0.041	−0.914	0.361
Perceived health	−4.296	0.672	−0.219	−6.397***	0.000
Chronic illness	0.200	0.751	0.010	0.266	0.790
Cohabitation	0.322	0.311	0.036	1.035	0.301
Perceived loneliness	0.853	0.479	0.081	1.783	0.075
Perceived isolation	5.364	0.504	0.497	10.642***	0.000
Perceived social support	−0.108	0.029	−0.121	−3.666***	0.000
**Step 5**					
Sex	−2.505	0.997	−0.072	−2.513*	0.012
Age	−0.076	0.037	−0.081	−2.079*	0.038
Marital status	−0.158	0.321	−0.014	−0.493	0.622
Education level	0.049	0.378	0.004	0.129	0.897
Monthly income	0.353	1.028	0.013	0.343	0.731
Employment status	−0.340	0.441	−0.030	−0.771	0.441
Perceived health	−1.497	0.634	−0.076	−2.360*	0.019
Chronic illness	0.270	0.659	−0.013	−0.410	0.682
Cohabitation	0.291	0.272	0.032	1.066	0.287
Perceived loneliness	0.617	0.421	0.059	1.466	0.143
Perceived isolation	3.716	0.462	0.344	8.047***	0.000
Perceived social support	−0.042	0.027	−0.047	−1.559	0.120
Psychiatric illness	3.196	0.556	0.173	5.752***	0.000
Insomnia	0.703	0.104	0.223	6.737***	0.000
Resilience	−0.200	0.034	−0.207	5.842***	0.000

*p < 0.05, **p < 0.01, ***p < 0.001.

Step 1 explained a significant portion of the variance [*F*_(4, 473)_ = 17.391, *MSE* = 12.850, *R*^2^ = 0.128, *p* < 0.001] in DASS-21, and indicated significant effects for sex and age but not civil status and education level. Step 2 explained additional variance but did not indicate significant model fit [*F*Δ _(2, 471)_ = 1.257 *MSE* = 12.84, *R*^2^Δ = 0.005, *R*^2^ = 0.133, *p* = 0.285]. Monthly income and employment status were not significantly associated with depression, anxiety, and stress symptoms. Step 3 explained additional variance [*F*Δ _(2, 469)_ =25.649, *MSE* = 12.219, *R*^2^Δ = 0.085, *R*^2^ = 0.218, *p* < 0.001] and indicated significant effects for self-perceived health status, but not the number of diagnosed chronic illnesses. Step 4 explained additional variance [*F*Δ _(4, 465)_ =77.885, MSE = 9.496, *R*^2^Δ = 0.314, *R*^2^ = 0.532, *p* < 0.001] and indicated significant effects for self-perceived isolation, perceived social support but not self-perceived loneliness and the number of cohabitants. Step 5 explained additional variance [*F*Δ _(3, 462)_ =48.842, *MSE* = 8.301, *R*^2^Δ = 0.113, *R*^2^ = 0.645, *p* < 0.001] and indicated significant effects for the number of diagnosed psychiatric disorders, insomnia, and resilience.

## Discussion

The main objective of this study was to analyze the psychosocial response to the COVID-19 pandemic in adults residing in Panama. Overall findings indicate several protective and risk factors associated with mental health outcomes for this sample of Panamanian adults during the COVID-19 pandemic. Social psychological factors, such as perceived social isolation (33, 34) and social support (35) accounted for the greatest proportion of the variance in depression, anxiety, and stress symptoms (36).

Our findings suggest that quarantine, isolation, and social distancing had a significant impact on the participants; more than half reported feeling affected by the COVID-19 pandemic, specifically regarding recreational activities and hobbies, social relations, mental health, and their income. These findings are in line with other recent studies showing that the biosafety measures implemented to stop the spread of the virus have significant implications for the psychosocial well-being of humans (37–39). Nevertheless, some people reported that during confinement they did not feel alone and that they were satisfied with the support of their loved ones. In this study, we reported negative relationships between the perception of social support and resilience on depression, anxiety, and stress symptoms. Similarly, several other studies have shown that social support, social well-being, prosocial behaviors, and resilience are factors that can enhance an adaptive response to stressful situations (40, 41). Thus, these findings point toward potential protective factors for individuals undergoing quarantine and lockdowns (42).

Regarding the risk factors for exposure to the virus, the current study documented a higher risk of infection among younger adults. Although older adults are a vulnerable group due to higher rates of chronic illnesses and increased mortality rates, emerging adults are more prone to contagion and spread of the virus due to social exposure and the belief that they are less at risk for severe symptoms (43–45). Moreover, other factors linked to the risk of contagion include having high exposure to the virus due to one's profession (e.g., healthcare worker), pregnancy, being immunosuppressed, smoking, and not following biosafety measures. Studies also indicate that vulnerable groups are affected by deficiencies, risks, or limitations related to health services, economic conditions, overcrowding, family dysfunction, unhealthy housing and environment, social insecurity, and discrimination. These risk factors increase the probability of comorbidities such as diabetes, obesity, hypertension, immunosuppression, or smoking (24, 25).

Furthermore, results indicated that male participants reported an increase in the consumption of cigarettes and alcoholic beverages. Some studies show that the stress derived from isolation can be a potential trigger for cigarette and alcohol consumption, which may indicate a maladaptive response to the pandemic (46, 47). In contrast, other studies documented that the pandemic encouraged some people to quit smoking, as smoking has been identified as a risk factor for more severe COVID-19 symptoms (48–51).

In line with other recent studies, results indicated that women experienced greater depression, anxiety, and stress symptoms than men (8–11). Sex differences in mental health symptoms are widely documented (52, 53) and recent research suggests that the COVID-19 pandemic uniquely affected the mental health of men and women. For example, many adult women experienced greater stress during confinement due to increased childcare demands and economic concerns (e.g., loss of employment, work from home mandates (54).

Our analyses also indicated that marital status, education, and economic factors (i.e., monthly income and employment status) were unrelated to depression, anxiety, and stress symptoms. Age and self-perceived health status—but not chronic illness—were negatively associated with depression, anxiety, and stress symptoms. Self-perceived social isolation was positively associated with depression, anxiety, and stress symptoms, whereas self-perceived social support was negatively associated with depression, anxiety, and stress symptoms. However, perceived loneliness and the number of cohabitants were unrelated to depression, anxiety, and stress symptoms. Psychiatric illness and insomnia were positively associated with depression, anxiety, and stress symptoms, whereas psychological resilience was negatively associated with depression, anxiety, and stress symptoms.

The results of this study corroborate previous research documenting an association between self-perceived health status and symptoms of depression, anxiety, and stress (55). Moreover, these results showed that chronic illnesses were not associated with symptoms of depression, anxiety, and stress (56, 57). One possibility for this finding is that most participants were young adults (aged 18–29) who had relatively few chronic illnesses. In addition, depression, anxiety, and stress was unrelated to the marital status, level of education, monthly income, and current employment status of participants. This further contradicts recent research documenting that lower education, low socioeconomic status, and unemployment is associated with greater symptoms of depression, anxiety, and stress (10, 58, 59). However, psychiatric illnesses were associated with symptoms of depression, anxiety, and stress. Feelings of loneliness and isolation are detrimental to mental health as they can be considered risk factors for the development of mental disorders such as depression, anxiety, adjustment disorder, chronic stress, insomnia, or dementia in old age (60, 61). Hence, preexisting mental health problems may be a notable risk factor for psychological distress during lockdowns (62).

In this study, young adults reported higher levels of depression, anxiety, and stress compared to adults. One study indicated that there were higher levels of stress, anxiety, and depression in adults aged 18 to 25 years compared to adults aged 26 to 60 years, and that people over 61 years old scored the lowest in stress, anxiety, and depression (56). Another study documented that people between 18-and 30 years old and over 60 years old presented higher levels of stress compared to middle aged adults (63). In contrast, other research documented that emerging adults experienced higher stress levels, whereas older adults experienced greater anxiety and depression (64). Additionally, women presented higher levels of depression, anxiety, and stress, similarly to other recent studies (65). Results also indicated a negative association between psychological resilience and symptoms of depression, anxiety, and stress. This corroborates previous research documenting that depression, anxiety, stress, insomnia, social disturbance, and somatic symptoms are associated with lower resilience (66). This finding may highlight the importance of resilience as a protective factor in the development of mental health problems in the context of pandemic lockdowns. Likewise, other factors, such as low income, familial problems, and less educational attainment may reduce individuals' resilience (67). In this sample, emerging adults scored lower in resilience compared to older adults. Previous research has indicated that emerging adults are affected more acutely by experiencing a loss or a traumatic situation, therefore, they may have difficulty understanding and controlling negative thoughts and unpleasant emotions such as fear, anger, irritability, and aggressiveness that arise due to social isolation and health-related stress (66, 68, 69).

## Limitations

This research employed a correlational and cross-sectional design, which prevents from drawing causal conclusions about the psychosocial effects of lockdowns. Participants were recruited *via* online convenience sampling, which constrained the pool of potential participants, thereby rendering the study's results as less generalizable. For instance, this study was limited to people with access to social media, computers, or smartphones, which may have resulted in the recruitment of participants who were younger, more educated, and more affluent than the general population. This sampling method may have also yielded a greater number of female participants because women are more interested and willing to participate in online psychological research than men (70). Indeed, several recent COVID-19 online survey studies that used similar recruitment methods obtained samples comprised of a disproportionate number of female participants (54, 71, 72). Nevertheless, at the time of the study, lockdown restrictions affected participants' and researchers' mobility, therefore online surveys were the only feasible option to collect data.

Despite the study's limitations, these data provide useful information about the mental health of Panamanian residents during the pandemic. The strengths of this study include the recruitment of a large sample and the utilization of valid instruments previously used and reported in similar studies. This study is the first report on the psychological impact of COVID-19 in the Panamanian general population and one of the only studies on the psychological impact of COVID-19 in the Latin American region, thus contributing to research on the Latin American population and low-middle income countries.

## Data availability statement

The raw data supporting the conclusions of this article will be made available by the authors, without undue reservation.

## Ethics statement

The studies involving human participants were reviewed and approved by National Research Bioethics Committee of Panama (Comité Nacional de Bioética de la Investigación, CNBI). The patients/participants provided their written informed consent to participate in this study.

## Author contributions

DCO conceived the study and manuscript. DCO and MSP wrote the manuscript. SR-A, AET, EP-Q, CC, CPR, and GBB read, reviewed, wrote sections and equally contributed to the intellectual content and format of the manuscript. All authors approved the submitted version.

## Funding

This work was supported by the Sistema Nacional de Investigación (SNI), the Secretaría Nacional de Ciencia, Tecnología e Innovación (SENACYT).

## Conflict of interest

The authors declare that the research was conducted in the absence of any commercial or financial relationships that could be construed as a potential conflict of interest.

## Publisher's note

All claims expressed in this article are solely those of the authors and do not necessarily represent those of their affiliated organizations, or those of the publisher, the editors and the reviewers. Any product that may be evaluated in this article, or claim that may be made by its manufacturer, is not guaranteed or endorsed by the publisher.
